# Design Strategies for Alkaline Exchange Membrane–Electrode Assemblies: Optimization for Fuel Cells and Electrolyzers

**DOI:** 10.3390/membranes11090686

**Published:** 2021-09-03

**Authors:** Aviv Ashdot, Mordechai Kattan, Anna Kitayev, Ervin Tal-Gutelmacher, Alina Amel, Miles Page

**Affiliations:** 1 Hydrolite Ltd., 2 Hatochen St., Caesaria 38900, Israel; aviv.ashdot@hydrolite-h2.com (A.A.); mor.kattan@hydrolite-h2.com (M.K.); anna.kitayev@hydrolite-h2.com (A.K.); ervin.tal-gutelmacher@hydrolite-h2.com (E.T.-G.); 2 Department of Chemistry, Bar Ilan Institute of Technology and Advanced Materials (BINA), Bar Ilan University, Ramat Gan 52900, Israel

**Keywords:** alkaline exchange membranes, fuel cells, electrolyzers, membrane–electrode assembly

## Abstract

Production of hydrocarbon-based, alkaline exchange, membrane–electrode assemblies (MEA’s) for fuel cells and electrolyzers is examined via catalyst-coated membrane (CCM) and gas-diffusion electrode (GDE) fabrication routes. The inability effectively to hot-press hydrocarbon-based ion-exchange polymers (ionomers) risks performance limitations due to poor interfacial contact, especially between GDE and membrane. The addition of an ionomeric interlayer is shown greatly to improve the intimacy of contact between GDE and membrane, as determined by ex situ through-plane MEA impedance measurements, indicated by a strong decrease in the frequency of the high-frequency zero phase angle of the complex impedance, and confirmed in situ with device performance tests. The best interfacial contact is achieved with CCM’s, with the contact impedance decreasing, and device performance increasing, in the order GDE >> GDE+Interlayer > CCM. The GDE+interlayer fabrication approach is further examined with respect to hydrogen crossover and alkaline membrane electrolyzer cell performance. An interlayer strongly reduces the rate of hydrogen crossover without strongly decreasing electrolyzer performance, while crosslinking the ionomeric layer further reduces the crossover rate though also limiting device performance. The approach can be applied and built upon to improve the design and production of alkaline, and more generally, hydrocarbon-based MEA’s and exchange membrane devices.

## 1. Introduction

Anion exchange membrane (AEM) devices have shown tremendous progress in the last few years [1]. High performance has been achieved in membrane–electrode assemblies of both fuel cells [2] and electrolyzers [3], although high-performing AEM electrolyzers currently require a feed of dilute alkaline electrolyte rather than deionized water. Similarly, commercially relevant durability has been shown for both fuel cells and electrolyzers. Hassan et al. recently showed more than 2000 hours of fuel cell durability at 75 °C in H_2_/O_2_ operation [2]. Hydrolite (formerly PO-CellTech) also reported >1000 hours in H_2_/CO_2_-free air in technical (250 cm^2^) cells at 67 °C [4]. Dioxide Materials meanwhile have reported over 10,000 hours in an alkaline water electrolyzer using Sustainion^®^ Grade-T membrane, at 60 °C and 1 A/cm^2^ current density [5]. These standout results herald significant steps towards demonstrating the full commercial potential of AEM technology. They are the culmination of community-wide efforts over the last two decades to identify and resolve the many scientific and technological challenges faced by AEM devices, the essence of which has been covered in several recent reviews [3,4,6,7].

The AEM has, perhaps, been subject to the heaviest focus due to known chemical stability challenges, and its central, heavy-duty role in the membrane–electrode assembly (MEA) [6,8,9,10,11,12]. From a performance point of view, anion conductivity and water transport are critical, while electrical isolation and gas separation are similarly important, especially when examining cell durability. These latter can be considered ‘mechanical’ components of the membrane function, provided primarily by ionomer backbones as well as possible additives [5], reinforcing structures [13,14,15,16], or crosslinking [17,18,19].

These mechanical considerations affect the boundaries of a fundamental trade-off between (a) maximizing water and ion conductance, both achieved (other things being equal) by decreasing membrane thickness and (b) minimizing gas crossover, pinholes, possible electrical shorting points, and membrane failure, all of which are mitigated by increased membrane thickness. The optimal points for these are clearly application-dependent: membranes of the order of 10 μm are preferred for fuel cells, mainly to maximize cross-membrane water transport [20], whereas electrolyzers demand much thicker membranes, especially if in situ electrochemical hydrogen pressurization is sought, where an order of magnitude closer to 50–100 μm may be appropriate with current technology [21,22]. In both cases, technological improvements in mechanical aspects of MEA fabrication could allow for thinner membranes.

Strong advances have been made towards resolving classically recognized technical challenges such as chemical degradation of AEM’s and recast alkaline ionomers [23], development of non-precious/non-Pt electrocatalysts [24], optimizing cell water management [25], optimizing GDL’s [26], etc. The nominally more mundane but essential task of successfully integrating the component layers into a mechanically robust, electrochemically high performing MEA has, however, not been well studied, although its impact on device performance and durability can be major [4,11].

**Figure 1:**
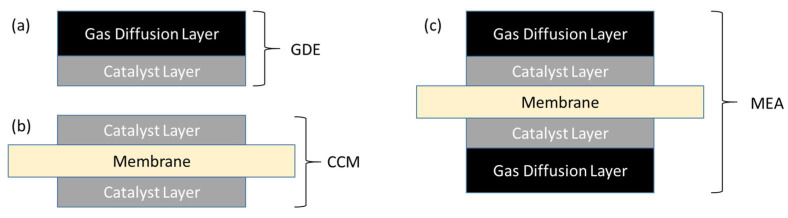
Schematic cross-sectional depiction of membrane–electrode assembly components: (**a**) gas diffusion electrode (GDE) and (**b**) catalyst-coated membrane (CCM) sub-components; (**c**) a fully assembled MEA from either GDE’s + Membrane or CCM + GDL’s.

The two most common methods of producing a typical, five-layer MEA are illustrated schematically in Figure 1. In the first case (Figure 1a), a catalyst ink consisting of catalyst particles and dispersed ionomer and possibly other additives is deposited on a GDL by spray-coating, screen- or roll printing, doctor-blade, or other method, to form a gas diffusion electrode (GDE). Two such layers form the anode and cathode electrodes, and are compressed either side of an exchange membrane to form the MEA. In the second case (Figure 1b), catalyst inks are deposited onto either side of the membrane to form a catalyst-coated membrane (CCM). The CCM is then sandwiched between two GDL’s to form a nominally identical MEA (Figure 1c).

As can be seen, two main interfaces are present on each side of the MEA: One between the GDL and the catalyst layer (CL), and a second between the CL and the membrane. A key challenge in the production of high-quality alkaline MEA’s, which are most commonly fabricated from hydrocarbon-based ionomers, is to achieve high-quality interfaces: Unlike common perfluorinated ionomers, hydrocarbon ionomers do not have an accessible glass transition, and so cannot be effectively fused by hot-pressing. This may lead to performance losses and degradation generated by an insufficiently intimate and/or progressively delaminating membrane/CL interface [27]. As we will show, the choice of selected production route of the MEA is a highly important technical consideration for AEM devices whereas in proton exchange membrane (PEM) devices, consideration may be determined more commonly by economics or convenience.

One approach to improved interface construction has been the development in recent years of the so-called direct membrane deposition (DMD) approach, applied to PEM [28,29], and recently also to AEM [30] fuel cells. Initially conceived to improve performance in PEM MEA’s via a lower achievable membrane thickness [31], the application to AEM’s is of interest both for this reason [20], as well as for the potential to achieve better interfacial contact with only one catalyst layer/GDL interface created during assembly. DMD generates impressive results, although the absence of a separately produced and quality controlled free-standing membrane can pose its own difficulties for commercial devices.

In this work we introduce the concept, borrowing in part from DMD-type MEA’s, of an ionomeric “interlayer” between catalyst layer and membrane, as a means to improve interfacial contact. We employ through-plane impedance measurements under controlled temperature and humidity to make quantitative comparisons of contact resistance between MEA’s fabricated via CCM and GDE methods. We examine the resulting fuel cell and electrolyzer performance and, with an eye on improving MEA’s for alkaline membrane electrolyzers, the effect of the interlayer approach on reducing the hydrogen crossover rate in the MEA’s, both as a function of interlayer thickness, and further by employing post-production crosslinking of the interlayers [32,33].

The interlayers are shown to improve both contact resistance between a GDE and membrane and hydrogen crossover rate, and the results further illustrate the importance of quantifying device performance losses arising from MEA production methods in hydrocarbon-based exchange membrane devices. The mechanically-focused measurements employed are shown to be good predictors of device performance. This allows new production engineering strategies for hydrocarbon-based ionomers, which can help compensate for the lack of an effective hot-pressing process, to be efficiently evaluated and optimized.

## 2. Materials and Methods

### 2.1. Materials

Catalysts: Carbon-supported platinum (Pt/C) catalyst (40% Pt on Vulcan XC-72) was purchased from Alfa Aesar (Haverhill, MA, USA) for use in hydrogen crossover experiments. Proprietary Hydrolite fuel cell electrodes and electrolyzer cathode catalysts were used for performance testing. Electrolyzer anodes were prepared with NiFe_2_O_4_ catalyst purchased from US Research Nanomaterials (Houston, TX, USA).

Gas diffusion layers: Fuel cell electrodes and electrolysis cathodes were from Freudenberg AG (Weinheim, Germany) type H23C6. Nickel fiber paper gas diffusion layers for electrolyzer anodes were purchased from Dioxide Materials (Boca Raton, FL, USA).

Ion exchange polymers (ionomers) are based on substituted polyarylene precursor polymers, which when fully functionalized with quaternized amine functional groups have a maximum Ion Exchange Capacity (IEC) of ~2 mmol·g^−1^.

Amination reactions: Trimethylamine (TMA, 50 *w*/*w*% in water) and N,N,N′,N′-tetramethyl-1,6-hexanediamine 99.8% (TMHDA) were purchased from Holland Moran(Yehud-Monosson, Israel).

### 2.2. Amination and Crosslinking

GDEs and membranes made with ionomer precursor were immersed in TMA/TMHDA baths at varying molar ratios of TMA to TMHDA, at a total concentration 0.8 M. The reaction was carried out in a fume hood over 15 hours at room temperature. In the next steps, the samples were washed thoroughly with de-ionized (DI) water and soaked in H_2_SO_4_ solution (1 M, 15 min) to terminate the amination process, washed again in DI water (15 min) and ion exchanged to carbonate form by washing three times in NaHCO_3_ (1 M, 15 min each wash). 

### 2.3. Catalyst Ink Preparation

For crossover and conductivity tests, identical Pt/C catalyst layers were used for anode and cathode. To prepare the catalyst inks, a 5 wt% dispersion of ionomer (in ethanol) or ionomer precursor (in tetrahydrofuran) was mixed with 40% Pt/C particles pre-wetted with DI water. Final inks contained 13.9 wt% ionomer/86.1 wt% Pt/C. The resulting polymer-catalyst slurry was homogenized with an ultrasonic probe for 15 min, while chilled in an ice bath.

GDEs and CCMs were fabricated by airbrush deposition of the catalyst ink onto a larger area substrate (membrane or GDL), from which 5 cm^2^ electrodes were cut. Electrodes for crossover tests were prepared with 0.3 mg/cm^2^ Pt loading.

### 2.4. Cell Production

CCM’s and GDE’s were prepared by spray coating onto a relevant substrate, held on a temperature-controlled vacuum table. Layers were assembled into MEA’s and placed with Teflon gaskets to achieve a 20–40% pinch on the membrane–electrode assembly. The assembly was sandwiched into 5 cm^2^ cell hardware (Fuel Cell Technologies, Albuquerque, NM, USA) with graphite polar plates containing serpentine flow fields. For the electrolyzer, stainless steel (316L) cell endplates were used in place of aluminum.

### 2.5. Interlayers

Interlayers were coated by airbrush on top of catalyst layers. Dispersions of 2.5 wt% ionomer in ethanol, or ionomer precursor in tetrahydrofuran, were sprayed onto ~50–100 cm^2^ GDE’s (area accurately known) while keeping the spray table surface in the range of 60–80 °C. Loadings were determined by drying then weighing the GDL substrates on an analytical balance before and after spray coating. Resulting electrodes were then cut down to required sizes for experimentation.

### 2.6. Membrane Casting

Dispersions of ionomer in HCO_3_^−^ form (5 wt% in ethanol), or precursor (5% in tetrahydrofuran), were poured into a flat, 15 cm diameter Pyrex petri dish. The petri dish was inserted carefully into a vacuum oven for drying (60 °C overnight).

Ionomer membranes were washed with 1 M KOH solution followed by DI water; the resulting membrane swelling allowed peeling away from the petri dish to yield free- standing membranes.

### 2.7. Ion Exchange Capacity Measurement

IEC was measured with potentiometric titration: Samples were converted into Cl^−^ counterion form by immersing in 1 M NaCl solution for 30 min, during which time the solution was refreshed at least three times. Samples were then rinsed thoroughly with DI water to remove any excess Cl^−^ ions. Samples in Cl^−^ form were ion-exchanged in a similar fashion using 0.2 M NaNO_3_ solution (30 min with at least four exchange solution changes), while collecting the exchange solutions (repeating four times). The Cl^−^ containing nitrate solutions were combined and titrated against a AgNO_3_ standard (0.01 M) using an Ag^+^ ion-selective electrode. The membrane was then exchanged back into Cl^−^ form, washed, dried in vacuum oven (50 °C, 3 h) and weighed. The IEC was calculated according to:

IEC = Δ*V*_AgNO3_·*C*_AgNO3_/*m*_d_, (1)

where *m*_d_ is the mass of the dry membrane (in the Cl^−^ form), Δ*V*_AgNO3_ is the consumed volume and C_AgNO3_ the concentration of the AgNO_3_ titration solution.

### 2.8. Fuel Cell Tests

Prior to assembly, GDE’s and membranes, or CCM’s (“ionomer-containing components”), were immersed in aqueous NaOH (3 M, 10 min), to ensure ion exchange of the membrane and ionomer, then washed thoroughly in DI water to remove any remaining NaOH. The cells were assembled into Scribner Associates fuel cell hardware (5 cm^2^ active area, single serpentine flow pattern), and closed with a torque of 7 N·m on each bolt. Kapton gaskets were used to achieve 20–40% pinch on the membrane electrode assembly. Humidified streams of H_2_ and CO_2_-free air were supplied to the cell at a flow rate of 0.10 L/min and 0.60 L/min for anode and cathode respectively. The cell was operated at 80 °C with a back pressure of 3 bar(g) and 1 bar(g) at the anode and cathode, respectively. Dew points were 65 °C and 75 °C in the anode and cathode gas feeds, respectively. Heated gas input lines were maintained 5 °C above the respective gas dew points. All of the polarization curves were run with a scan rate of 10 mV/s.

### 2.9. Electrolyzer Tests

1 M KOH solution was flowed through anode and cathode at a flow rate of 500 mL/h. The cell and electrolyte were heated to 80 °C over ~30 min, and polarization curves recorded from 1.4–2.2 V, with a scan rate of 10 mV/s.

### 2.10. Hydrogen Crossover Tests

Hydrogen crossover was measured electrochemically using linear sweep voltammetry [34]. Ionomer-infused membrane supports with a thickness of 5 μm were sandwiched between cathode and anode GDEs. The MEA’s were assembled in 5 cm^2^ fuel cell hardware and tested in a Scribner Associates 850E Fuel Cell Test Station equipped with a potentiostat. Fully humidified hydrogen and nitrogen were fed to the counter and working electrodes respectively. Back pressure was set to 3 bar(g) (counter, H_2_) and 1 bar(g) (working, N_2_) providing a 2 bar pressure differential. The cells were held under gas flow until a stable OCV of *ca*. 0.1 V was observed. Linear sweep voltammograms were then conducted from 0.0–0.8 V. The current density plateau reached at positive voltage is taken as the hydrogen crossover rate in units of limiting current density.

### 2.11. Conductivity Measurements

Through-plane conductivity measurements were performed using a Scribner Associates MTS740 Membrane Test Station to collect Electrochemical Impedance Spectra at controlled temperature and relative humidity. Potentiostatic impedance was measured under alternating potential of amplitude 10 mV from 10 MHz to 1 Hz. The high-frequency intersection with the real axis represents the ohmic high-frequency resistance (HFR). All measurements were performed under N_2_ environment in controlled humidity and temperature conditions. Samples were 0.5 cm^2^ in active area and were loaded in bicarbonate ionomeric form to avoid an uncertain degree of carbonation during testing.

### 2.12. HR-SEM Imaging

SEM image was taken in high-vacuum mode at an acceleration voltage of 4 kV using a secondary electron detector with a Zeiss Ultra-Plus FEG Scanning Electron Microscope (Zeiss Microscopy, Jena, Germany).

## 3. Results

**Figure 2:**
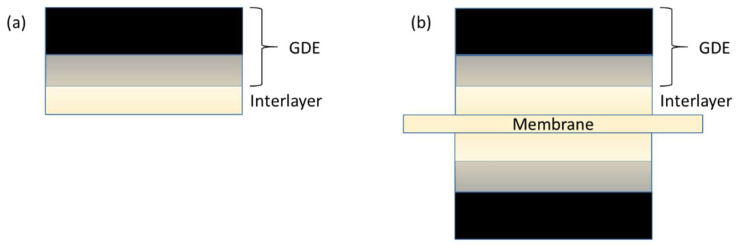
Schematic of the gas diffusion electrode + interlayer approach to membrane–electrode assemblies. The interlayer is deposited by casting an ionomer dispersion onto the gas-diffusion electrode (**a**), thereby creating a relatively high-quality ionomer–CL interface. An MEA is then assembled by sandwiching a membrane between two such gas-diffusion electrodes (**b**).

The “GDE + Interlayer” concept is illustrated in Figure 2. An ionomeric layer was applied to a prefabricated GDE (Figure 2a). Anode and/or Cathode GDE’s with interlayer were assembled with a membrane to yield a seven-layer (Figure 2b) or six-layer MEA, respectively.

The interface between GDL and catalyst layer is, of course, also significant since any surface roughness will increase cell HFR [35] and leave cavities that may also allow for losses and/or degradation due to water accumulation [36]. However, being an electronic interface with orders of magnitude lower resistivity than the ionic interface, it is less acute than the second, ionic interface between CL and membrane. 

In the GDE + interlayer approach, the burden of providing intimate ionic contact between the membrane and electrode is transferred from the predominantly nanoparticle-based catalyst layer, with a relatively small proportion of dispersed, recast ionomer, to the purely ionomeric layer which possesses a relatively intimate contact, created a priori during the interlayer casting process, with said catalyst layer.

We begin with an examination of the differences between CCM and GDE approaches, and the effect of employing an ionomeric interlayer in the GDE, with respect to interfacial impedance and fuel cell performance. We subsequently examine the membrane-like qualities of the interlayers, with and without post-production crosslinking, with evaluation in terms of hydrogen crossover and electrolyzer performance.

### 3.1. Membrane-CL Interfacial Resistance Measurements

**Figure 3:**
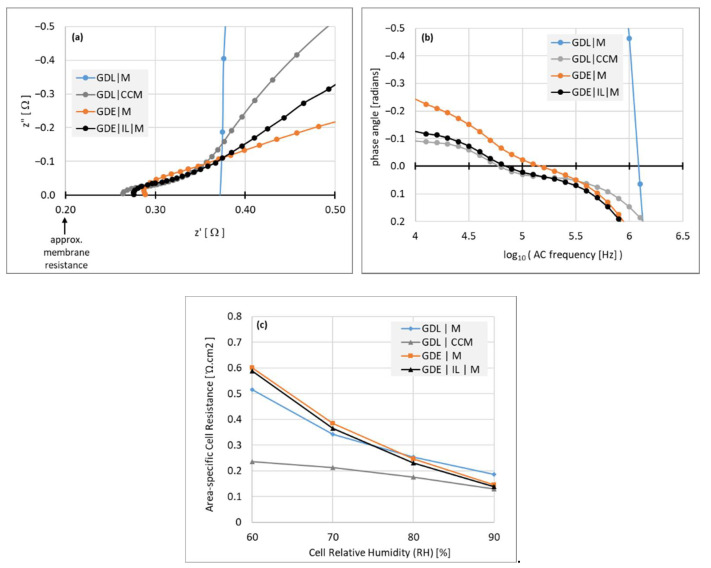
Interfacial resistance data from through-plane measurements of impedance of different MEA construction methods, taken at T = 80 °C in bicarbonate form. (**a**) Nyquist plots, z” vs z’, taken at 90% RH; (**b**) the same data set, plotting phase angle of the alternating current (AC) against AC frequency; (**c**) area-specific resistance isotherms at varying RH from 60 to 90%.

Through-plane impedance measurements, shown in Figure 3, were used to compare the quality of GDL|CL|Membrane interfaces of model MEA’s, generated by these different assembly routes. Each MEA consisted of a ~20 μm membrane, between Pt/C catalyst layers and GDL’s with a microporous layer.

Through-plane impedance experiments are typically used to measure membrane conductivity. The membrane is sandwiched between two standard GDL’s, giving a GDL-membrane interface with a known, or separately measured, contact resistance. This contact resistance is then subtracted out to generate a membrane conductance, which when normalized for membrane thickness yields a conductivity that should match an in-plane measurement that is insensitive to contact resistance.

In the measurements below however, no contact resistance subtraction was made. Instead, we evaluated the total resistance from the GDL to GDL, with different approaches to making the contact according to the MEA production approach. We assumed that resistances between test station electrodes and the GDL’s, and between any two electronically conductive layers, were low relative to interfacial resistances involving ionic contact. The measurements thus yield the sum of ionic–ionic interfacial resistances, ionic–electronic interfacial resistances, and membrane ionic resistance.

As the membrane is identical in each sample, the variation in the resistance measurement determines the variation in the sum of these ionic interfacial resistances in each sample assembly. It is noted that this high-frequency resistance is not sufficient to estimate device losses resulting from the interfaces, which is a DC resistance that incorporates the charge transfer elements of the interfacial impedance. This point will be addressed further in the Discussion section.

Assemblies were fabricated by different routes, depicted by the layer sequence of one side: The CCM route (GDL | CCM), GDE route (GDE | M, where “M” depicts the membrane), GDE route with added ionomeric interlayer (GDE | IL | M, where “IL” depicts the interlayer), and with a naked membrane–GDL assembly for reference, (GDL | M). The measurements were taken at 80 °C, with the ionomer in bicarbonate form to avoid questions of carbonation of hydroxide counterions.

The total HFR including membrane resistance and the sum of contact resistances was measured at the high-frequency point of zero phase angle, where z” = 0 in the Nyquist plots (Figure 3a). It can be seen therefore that all the electrode assembly variations show significantly lower contact resistance than that of the bare GDL–membrane. The assemblies themselves vary in the order (GDL | CCM) < (GDE | IL | M) < (GDE | M).

Figure 3b shows plots of the AC phase angle against frequency, where the electrode assemblies reach the zero phase angle point at a frequency approximately an order of magnitude lower than that of the bare (GDL | M) assembly, the shorter timescale of the interfacial capacitance indicating more intimate contact. Figure 3c shows isotherms of the cell resistance at varying RH from 90% down to 60%, where it can be seen that only the (GDL | CCM) assembly displays satisfactory performance as humidity decreases. This somewhat surprising result can be better understood with reference to a capacitive interface and examining the impedance data further, as addressed in the Discussion section.

### 3.2. Imaging the CL | IL Interface

**Figure 4:**
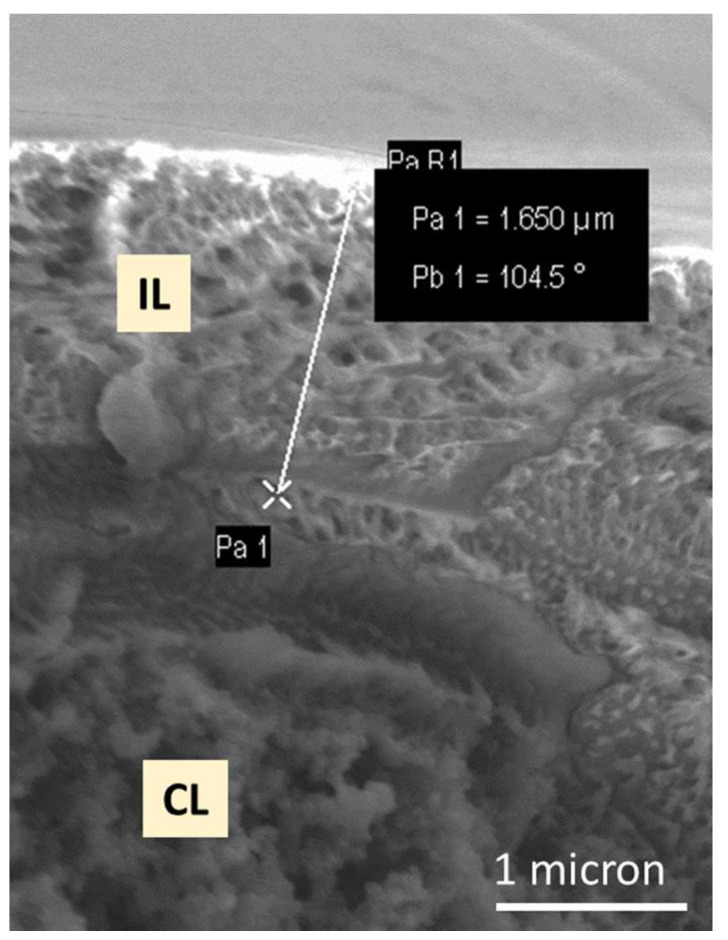
Cross-sectional SEM image of an ionomer interlayer (IL)/catalyst layer (CL) interface, showing the ca. 1.7 μm interlayer protruding from the catalyst layer.

Figure 4 is an SEM image of the cross-section of a GDE onto which 0.5 mg/cm^2^ of ionomer has been deposited. Areas that are predominantly interlayer and catalyst layer can be observed, together with a somewhat intermixed interface region between the two layers.

### 3.3. Performance of MEA’s in Fuel Cell Operation

**Figure 5:**
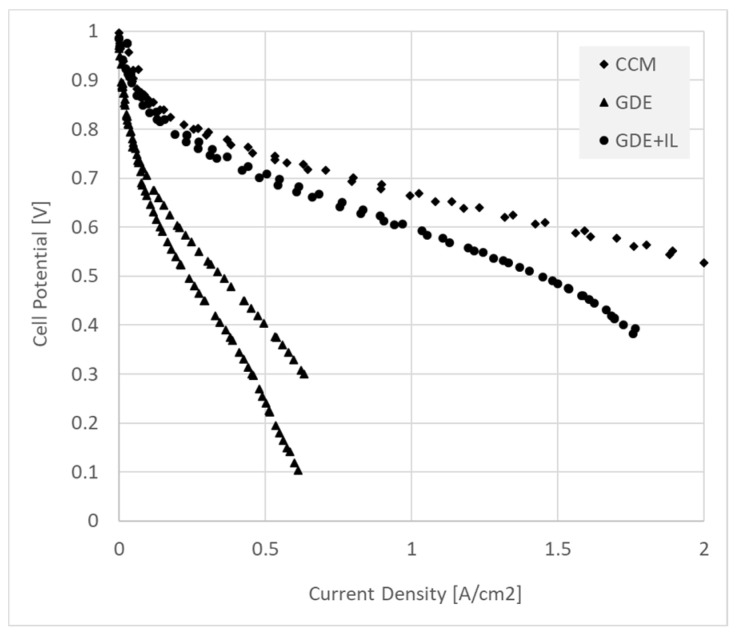
Polarization curves recorded in H_2_/CO_2_-free air for a 5 cm^2^ fuel cell MEA’s made from CCM’s (diamonds), GDE’s (triangles), and GDE’s with interlayer (circles). Note that the GDE form showed very poor relative performance, whereas adding an interlayer (~0.1 mg/cm^2^ of ionomer) allowed performance approaching that of the CCM.

Figure 5 shows a comparison of fuel cell performance for MEA’s fabricated with GDE (with and without interlayer) and CCM data. Conditions are described in the Methods section. The fuel cell data show the same trend as the contact resistance, with the CCM’s displaying the best performance, while adding an interlayer substantially improved GDE performance.

### 3.4. Cross-Linking of Ionomer Precursors via Quaternization of Secondary Diamines

**Figure 6:**
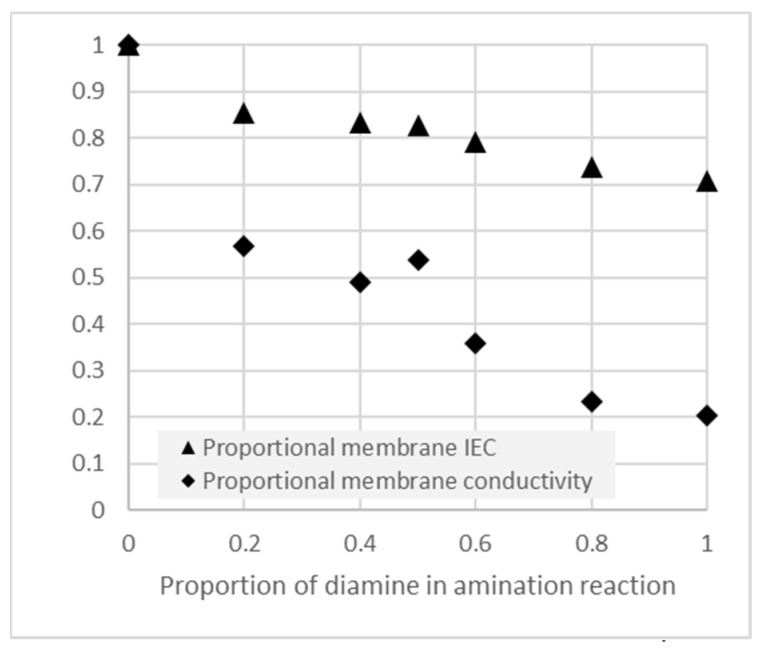
IEC and conductivity of crosslinked AEM’s, as a proportion of the uncrosslinked values, following amination reactions with varying proportions of the crosslinking diamine (TMHDA) together with the non-crosslinking monoamine (TMA).

The material properties of recast ionomer within a catalyst layer are inherently difficult to evaluate due to their low concentration relative to the catalyst and other solid components. Therefore, free-standing membranes of the polymer precursor were cast and aminated in TMA/TMHDA solutions. Following completion of the amination reaction, changes to IEC and conductivity were evaluated, as set out in Figure 6.

Noting that the outcome of the amination reaction may be quantitatively different in the catalyst layers, we nevertheless used these properties as a qualitative guide to the expected outcome in the catalyst layers, as well as in the membrane-like interlayer deposited on the catalyst layers.

It can be seen in Figure 6 that increasing the concentration of the crosslinking reactant lead to a lower degree of overall amination (lower achieved IEC) as well as a strong loss of anion conductivity in the layer. Such crosslinking would therefore need to be carefully controlled to optimize the tradeoff between performance and the intended cross-linking functionality.

### 3.5. Hydrogen Crossover in GDE-Based MEA’s 

In the electrolyzer, the ionomer–CL interface contact resistance is relatively very low, and GDE-type MEA performance is quite high, since for these experiments the electrolyzer device operates in 1M aqueous KOH. Any “gaps” at the interface will have the high ionic conductivity of the electrolyte solution (in the hundreds of mS/cm) and, in addition, the ionomer is kept well-hydrated and thus conductive.

Such gaps are, however, potentially problematic as they may act as pooling points for gaseous hydrogen and oxygen (on respective sides), and which, especially under pressure, may facilitate gas crossover, pinhole development, may form “hot spots”, etc., and would, therefore, best be minimized. With this in mind, we measured the performance of interlayers in terms of mitigation of hydrogen crossover. As the interlayers formed at least a semi-continuous layer over the GDE, they have a membrane-like quality that was optimized by minimizing hydrogen crossover versus ‘membrane thickness’. In the case of the interlayers, this thickness is not well-defined, and we used instead a value of ionomer loading (mass of ionomer per cm^2^ of electrode) in the interlayer. Here the hydrogen crossover rate was measured in units of A/cm^2^ of limiting current in the opposite electrode (see Section 2).

Optimization was performed by fabricating GDE’s and interlayers with ionomer precursors, which were crosslinked to varying degrees during a post-production quaternization reaction as described previously [33]. We do not claim knowledge of the quantitative degree of crosslinking, but following the experiments above (Figure 6), we made the presumption that the reaction proceeded correctly. Based on conductivity and crossover results, we also surmised that an unknown but increasing amount of crosslinking occurred with increasing proportions of TMHDA in the amination reaction bath.

**Figure 7:**
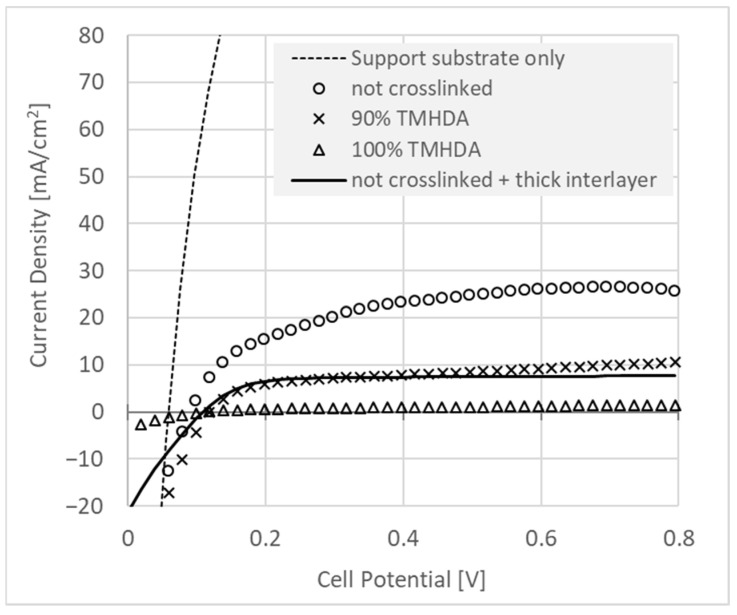
Hydrogen crossover measurement versus layer thickness. For this experiment, an ultra-thin (5 μm) reinforced, ionomer-infused support substrate was used as a dummy membrane which provided ionic conductivity between counter and working electrodes without substantially inhibiting hydrogen crossover. This substrate was placed between identical GDE’s, allowing a qualitative evaluation of the membrane-like properties of the interlayers. The crossover of the “support substrate only” (dashed line) was measured with interlayer-free GDE’s. Interlayer loadings are 0.12 mg/cm^2^, corresponding to <1 micron of interlayer, or 0.5 mg/cm^2^ (“thick interlayer”, solid line), on each electrode.

Figure 7 shows the effect on hydrogen crossover of adding interlayers with a selection of different thicknesses and crosslinking proportions. It can be seen that the thin (0.12 mg/cm^2^) membrane-like interlayer was able to significantly reduce crossover current. The combined amount of added ionomer from the two electrodes would be sufficient only for around 2 μm of equivalent membrane thickness. Adding cross-linking diamine (9:1 TMHDA:TMA) to the amination process further reduced the crossover current by a factor of more than two. Note that the using 100% TMHDA in the amination process yielded data that do not show sufficient current density in the cathodic direction, where hydrogen evolution should occur. It is possible then that the limiting current in the anodic direction, which should be determined by hydrogen crossover rate, may instead result from cell pathology, and so this crossover result is not considered further. Finally, increasing the interlayer loading to 5 mg/cm^2^ on each electrode had a similar effect on the crossover rate as crosslinking the interlayer with the 9:1 TMHDA:TMA amination procedure.

### 3.6. Performance of MEA’s in Electrolyzer Operation

**Figure 8:**
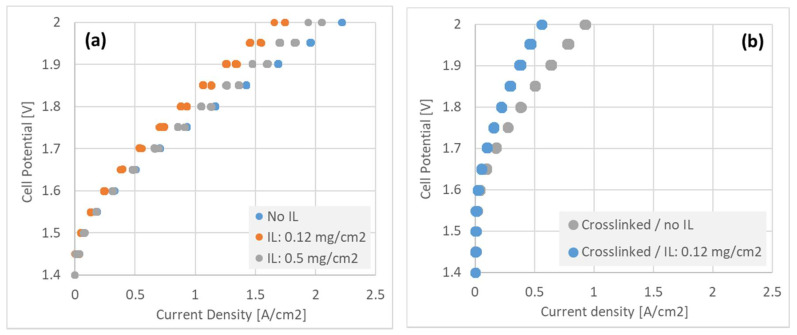
Polarization curves recorded for 5 cm^2^ electrolyzer MEA’s with various configurations of GDE + interlayer, incorporating (**a**) uncrosslinked and (**b**) crosslinked (90:10 TMHDA:TMA amine ratio) ionomer.

Figure 8 shows electrolyzer polarization curves at 80 °C under circulation of 1 M KOH in both electrodes, with different interlayer configurations at the cathode. Figure 8a shows non-crosslinked interlayers of increasing thickness. The GDE with no interlayer showed the highest performance, whereas the thick (0.5 mg/cm^2^) interlayer performed slightly better than the thin (0.12 mg/cm^2^) layer. The difference in performance between these three, non-crosslinked GDE’s was moderate however, allowing for co-optimization of crossover and performance by interlayer loading.

The crosslinked GDE’s on the other hand (Figure 8b) showed relatively poor performance with only approximately 1 A/cm^2^ achievable at 2.0 V, and much less when just the thin interlayer was added. It is clear that further optimization is needed on electrode crosslinking to improve electrolyzer performance, to enable the significant benefit to hydrogen crossover observed in Figure 7 to be exploited.

## 4. Discussion

### 4.1. MEA Preparation Methods, Interfacial Resistance and Performance

Important differences and limitations emerge between CCM and GDE-type production routes. Surface roughness ensures that the effective contact area is different from the geometric area, and the ratio between these two (which we do not attempt to quantify here) could be referred to as the “intimacy” of the contact. This value would be 1 for contact between two perfectly flat surfaces, while between rough surfaces, the ratio may vary strongly, from <<1 to >>1, depending on the quality of the interface formed.

**Figure 9:**
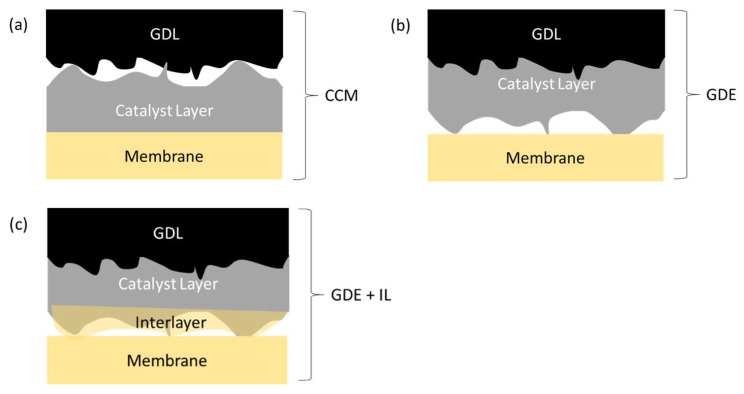
Schematic close-up of cross-sections of non-hot-pressed MEA’s generated by (**a**) CCM (**b**) GDE and (**c**) GDE + interlayer (“IL”) methods, illustrating the likely imperfections in the desired intimate contact between each of the layer interfaces. Note that the membrane surface, consisting of recast polymer (ionomer), can typically be made with lower roughness than GDL or CL surface consisting of stacked nanoparticles.

It is expected that interfaces established during production, by casting of one layer onto another, would be more intimate than those created during assembly, by physically pressing one to the other, since casting layers (of catalyst and/or ionomer) is done from a liquid that can spread to match the shape of the substrate layer, while simple mechanical pressing (not including hot-pressing, which is unavailable for ionomers used in this study) cannot achieve the same intimacy. The schematic in Figure 9 represents a closer view of the interfaces thus formed in CCM and GDE modes of MEA production. As we shall see, the results from Figure 3 can be interpreted on this basis.

Interfaces between more resistive layers, with lower contact area-specific conductance, would be more adversely affected by low effective contact area. In Figure 3a, the lowest contact resistance is indeed seen for the CCM, for which the ionomer–catalyst layer contact is the most intimate (Figure 9a), whereas this same interface is less intimate in the GDE (Figure 9b). A low interfacial impedance is less important in the (GDL | CL) interface, because it involves electronic resistivity, which for both the GDL and any well-designed catalyst layer, is orders of magnitude lower than the ionic resistivity.

The addition of the interlayer (Figure 9c), cast onto the GDE, decreases the overall resistance across the MEA (Figure 3a). This indicates that the improvement in the contact resistance due to greater intimacy is substantially greater than any increase in resistance due to increased overall ionomer thickness (counting the membrane + interlayer).

Some mechanistic information can be elucidated from Figure 3b, which shows the variation in the frequency at which the HFR value is reached. Note that an electronic–ionic interface as depicted in Figure 2 is expected to show RC-type equivalent circuit behavior in an impedance spectrum. The time constant, *τ*, of the element (the reciprocal of which determines the “high frequency” point) is directly proportional to the micro-structural effective surface area of the capacitive element for which capacitance C, proportional to *τ*, is related to the interfacial area A in the general capacitor equation:

(2) $$C=\frac{\varepsilon \varepsilon_{0}\times A}{d}$$

Since *A* represents the effective, rather than geometrical, contact area, increased effective ionic–electronic contact area provided by CL’s manifests itself as an increase in *τ*, and a decrease in the frequency at which the high-frequency zero phase angle occurs.

That same effective contact area is also a coefficient in the R term (the charge transfer resistance) of the RC element. This value of R, which is inversely proportional to *A*, contributes in series with the HFR to the DC resistance of the MEA, which is the relevant resistance in device operation. The relative frequency of the HFR is thus correlated to the DC resistance via A and provides a quite sensitive indication of the quality of the membrane–catalyst layer interface that is missed by the HFR measurement alone.

A dispersion of the two phases strongly increases the time constant versus the (GDL | M) configuration (Figure 3b), as well as decreasing the HFR. In a well-humidified MEA, where the ionomer is highly conductive (*ca*. 20 S·cm^−1^ in bicarbonate form, and about 5x greater in hydroxide form [37]), Figure 3b shows that all the catalyst layers provide a substantially improved intimacy versus a naked (GDL | M) interface, with an order of magnitude decrease in the high-frequency point of the impedance spectrum for the GDE, and even more for the CCM configuration. The addition of the interlayer to the GDE also brings the high-frequency point very close to that of the CCM.

Due to the better ionic contact achieved between membrane and CL, the CCM is still preferred for the fuel cell. However, CCM’s are not always straightforward to employ, especially in technical-sized AEM devices (active areas nominally greater than about 50 cm^2^ [38]). Membranes may potentially swell or even disintegrate when exposed to solvents such as those used in catalyst inks, especially if the ink is used to disperse the same ionomer as found in the membrane. Since electrode properties are strongly influenced by the ink solvents, compromising in this area to accommodate the membrane is undesirable and possibly even unfeasible, and a GDE-based approach would then be required.

The interlayer on the GDE goes some way to alleviating this issue. However, as can be seen in Figure 3c, when the MEA’s are dried to 60% RH, the (GDE | M) and the (GDE | IL | M) MEA’s both lose conductance, even to a greater extent than the naked (GDL | M) layer. This effect probably arises simply because of the strong loss of ionic conductivity due to low humidity: Any intimacy provided between ionomer and catalyst particles in the GDE (whether by ionomer already in the layer, or by the added ionomer interlayer) is nullified when the ionic conductivity is low, and overcome by the inherent roughness of the electronically conducting phase of the catalyst layer. 

The point of transition between these regimes is at about 75% RH (in Figure 3c), though this would vary according to the intrinsic conductivity, water uptake isotherm and especially degree of hydroxylation, of the ionomer in question. Thus even with an interlayer, the GDE approach appears to be more susceptible to dry conditions than the CCM and is thus (so far) only a partial solution, at least for fuel cells, that should benefit from further optimization of MEA production processes.

### 4.2. Fuel Cell Performance versus MEA Fabrication Process

As discussed above, the measured HFR provides only a hint of the effect of contact impedance on device performance in DC (e.g., polarization or constant discharge) measurements. This can be appreciated from the fuel cell polarization curves of Figure 5. Clearly, qualitative agreement between contact resistance (Figure 3a) and fuel cell device performance (Figure 5) is observed. The few mΩ·cm^2^ of additional contact HFR measured for the GDE cannot account for the performance loss measured in the device, even with the interlayer added.

The present study does not allow deduction of the absolute value of DC contact resistances from the MEA’s, since it cannot be distinguished from other losses in fuel cell operation. However, an estimation of the relative DC contact resistances between the different MEA fabrication methods may be made by determining and comparing the slope of the polarization curves in the pseudo-linear region, observed at intermediate current densities for each MEA type. These values (labelled *DCR*) and the HFR for each device are presented in Table 1. From these values, two contact resistances are determined, *relative to the CCM*, as follows (also shown in Table 1). The simplest model of contact impedance between imperfect surfaces, is used [39]. A series resistance, *R*_S_, is combined with a single RC equivalent circuit element, with resistive component *R*_1_. Given identical layers in the different MEA constructions, these values can be determined relative to a reference MEA (the CCM). The change in HFR then gives the relative *R*_S_, and the change in the DCR (with HFR subtracted out) gives the relative *R*_1_:

*R*_S_ − *R*_S,CCM_ ≈ HFR − HFR_CCM_, (3)

*R*_1_ − *R*_1,CCM_ ≈ DC resistance − (*R*_S_ − *R*_CCM_) (4)

The large change in fuel cell HFR for the GDE versus CCM is anomalous and suggests that additional effects come in to play under current, as a result of the relatively poor interface, that prevents further interpretation in the context of derived, apparent DC resistance.

Meanwhile the change from CCM to GDE+interlayer is in the range expected, based on the ex situ data (Figure 3a), given the shift from bicarbonate to hydroxyl form of the ionomer. We can then interpret the relative *R*_1_ increase as a contact impedance effect representing the performance cost of working with the GDE versus the CCM, with the mitigating interlayer in place.

It can be seen that the contact resistance at high frequency, as determined by both the ex situ and device tests, is comparable with the best available in the literature (see, for example, [2,25]), for both CCM and GDE with interlayer. Around 30% of the overall cell high-frequency resistance can be attributed to contact and not membrane resistance (Figure 3a and Table 1). However, the huge effect on the fuel cell performance (Figure 5 and Table 1), accompanied by a relatively minor effect on ex situ contact resistance, of a poor GDE interface is striking. The high frequency measure takes into account only the contact between membrane surface and electronically conducting catalyst layer. Ionomer in the catalyst layer of this MEA is insufficiently continuous with the layer–membrane interface under fuel cell conditions (including direct current and, potentially, low ionomer hydration at least in the cathode [20,36]). Low ionomer hydration is particularly damaging in hydrocarbon ionomers due to the highly sensitive dependence of conductivity on hydration [40].

In the CCM and GDE with interlayer, that continuity is strongly enhanced as seen in Figure 5. The more sensitive ex situ predictor of that success is the frequency at which zero phase angle is achieved (Figure 3b). It seems clear meanwhile, when a hydrocarbon-based ionomer is used, that contact impedance is very significant, and a full appreciation of this loss mechanism is of high importance in the construction of AEM Fuel Cell MEA’s.

### 4.3. GDE + Interlayer—The Effect on Hydrogen Crossover and Performance of Electrolysis Cells

The effect of the interlayer, including degree of crosslinking, on the H_2_ crossover is of primary concern for electrolyzer systems. It is desirable to be able to run these systems in highly pressurized environment, over 30 and even up to 100 bar, while maintaining a reasonably thin AEM for performance considerations. While the existence or not of an interlayer in the GDE cell has relatively little effect on performance (Figure 8a) as compared to the fuel cell configuration, the crosslinking process does adversely affect electrolyzer performance (Figure 8b). While we expect that optimizing the crosslinking process could mitigate this effect, it was observed meanwhile that a relatively thick interlayer, without crosslinking, had a relatively small effect on electrolyzer performance while greatly reducing membrane crossover. This apparent ‘sweet spot’ is highlighted in Table 2, which collates hydrogen crossover and electrolyzer performance of the various interlayer configurations.

This membrane-like effect of the interlayers is quite significant. It is perhaps dangerous to derive a value of specific hydrogen permeability, since the experiments are performed on an ionomer-coated electrode for which the thickness is not precisely known, but one can estimate a nominal equivalent membrane thickness from the ionomer loading and density: Assigning a nominal material density of 1 g/cm^3^, 0.12 mg/cm^2^ yields 2.4 μm worth of membrane (summing of the two electrodes).

In Table 2, the corresponding value for the non-crosslinked membrane of 26.6 mA/cm^2^ yields 1.6 × 10^−14^ mol·m^−1^·s^−1^·Pa^−1^. This value is comparable to a Nafion^®^ 117 membrane [41], although the material is by no means fully contiguous, with a large portion “soaking in” to the GDE, and no special effort yet made to achieve continuity. Indeed, the SEM image in Figure 4 of a 0.5 mg/cm^2^ interlayer shows ~2 μm thickness. Using this value instead of calculating a nominal estimated thickness, two electrodes (4 μm) gives ~8 mA/cm^2^ of crossover, translating to 4 × 10^−15^ mol·m^−1^·s^−1^·Pa^−1^, which is well under the crossover rate of even dry Nafion^®^ 117 membrane at the same temperature [42].

## 5. Conclusions

This work highlights the importance of the method of fabrication of alkaline membrane device MEA’s, and is likely generalizable to most hydrocarbon-based exchange membrane devices. By measuring and comparing the relative contact resistance of different fabrication approaches, it shows the lack of intimacy of contact that can arise if the MEA fabrication process is inadequate, as well as demonstrating that the commonly used high-frequency resistance measurement alone is insufficient to diagnose such a problem. Based on these results, we showed that adding an ionomeric interlayer to gas diffusion electrodes, which otherwise show poor interfacial contact to the membrane in an MEA, greatly mitigates (without completely solving) this ‘intimacy-of-contact’ problem. We also looked at exploiting such an interlayer approach for the production of alkaline water electrolyzer MEA’s, as a means to improve gas separation performance, and examine the possibility of optimizing this effect by in situ crosslinking. It was found that the interlayer was very effective, especially with crosslinking, in reducing hydrogen crossover, although the crosslinking in this work was quite detrimental to electrolyzer performance.

Useful development based on the results presented here could include, firstly, efforts to improve the intimacy of contact achieved by ionomer interlayers in GDE-based MEA’s, making use of ex situ frequency-resolved impedance measurement to predict likely success in the device. Optimizing performance of crosslinked interlayers also seems a low-hanging fruit, for example by modifying the degree of crosslinking and choice of crosslinking agents, as well as optimizing post-production reaction conditions. The approach taken here shows highly promising crossover results that could allow for thinner membranes in electrolyzers if the electrochemical performance can be improved. 

Finally, quantifying high pressure gas crossover of alkaline membranes sandwiched between interlayer-reinforced gas diffusion electrodes should be carried out, as well as evaluating the durability of MEA’s produced using these techniques in fuel cell and electrolysis devices.

## Figures and Tables

**Table 1:** Cell HFR, estimated DC resistance (~DCR) and derived estimates of contact resistances, relative to that of the CCM-based MEA, for the different fabrication methods (see text for further explanation). Note the cell HFR values do not match the ex situ impedance measurements (Figure 3) quantitatively because the fuel cell operates with membrane and CLs in hydroxide form, while the MTS measurements were performed in HCO_3_^−^ form.

MEA	HFR	~DCR	~(*R*_S_ − *R*_S,CCM_)	~(*R*_1_ − *R*_1,CCM_)	
CCM	42	140	0	0	(mΩ·cm^2^)
GDE	310	630	270	230	
GDE + IL	50	200	8	60	

**Table 2:** The hydrogen crossover (expressed as the limiting current density in the crossover experiment, in mA/cm^2^) and electrolyzer cell performance (current density at 1.8 V) are tabulated for different GDE+IL configurations.

MEA (GDL+IL)	H_2_ Crossover at ΔP = 2 bar (mA/cm^2^)	EL Current Density at 1.8 V (A/cm^2^)
IL 0.12 mg/cm^2^; Non-XL	26.6	0.88
IL 0.12 mg/cm^2^; 90% TMHDA	10.5	0.22
IL 0.12 mg/cm^2^; 100% TMHDA	--	--
IL 0.5 mg/cm^2^; Non-XL	7.7	1.05
IL 0.5 mg/cm^2^; 90% TMHDA	--	0.034

## Data Availability

The data presented in this study are available as supplementary materials.

## Supplementary Materials

The following are available online at https://www.mdpi.com/article/10.3390/membranes11090686/s1.

## Abbreviations

AEM	Alkaline Exchange Membrane
AC	Alternating Current
CCM	Catalyst-Coated Membrane
CL	Catalyst Layer
DC	Direct Current
DCR	Resistance in Direct Current
DI	De-ionized [water]
DMD	Direct Membrane Deposition
GDE	Gas Diffusion Electrode
HFR	High-Frequency Resistance
IEC	Ion Exchange Capacity
IL	Interlayer
MEA	Membrane Electrode Assembly
PEM	Proton Exchange Membrane
Pt/C	Carbon-supported Platinum
TMA	Trimethylamine
TMHDA	N,N,N′,N′-tetramethyl-1,6-hexanediamine
X | Y	Assembly of pre-fabricated MEA sub-components X and Y, where ‘|’ represents the interface created at time of assembly
